# Supplementary material for: Immunogenicity and Protective Efficacy of Five Vaccines Against Highly Pathogenic Avian Influenza Virus H5N1, Clade 2.3.4.4b, in Fattening Geese
Source: Vaccines (Basel). 2025 Apr 11;13(4):399. doi: 10.3390/vaccines13040399 (PMC12031072; doi:10.3390/vaccines13040399)
Supplement: Supplementary file 1 [file vaccines-13-00399-s001.zip › Figure S1.pdf]

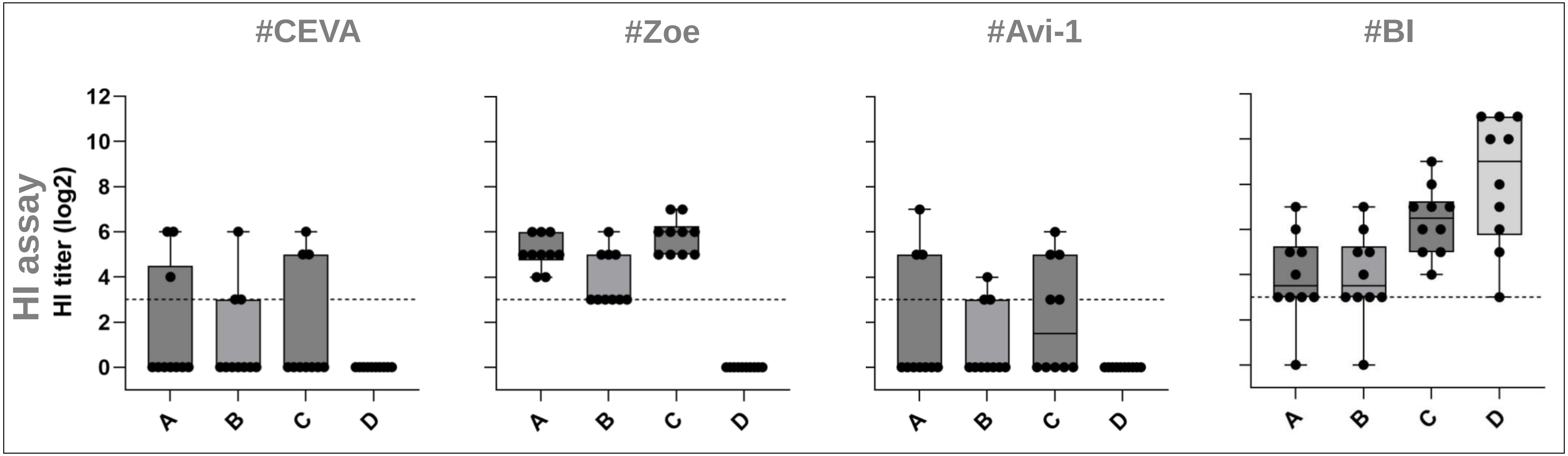

Figure S1. Plasma samples from 4 vaccine groups after prime immunization with positive HI results (>3 log_2]) in the HI assay against the Clade 2.3.4.4b homologous antigen (H5N1 A/chicken/DE-NI/AI 4286/2022 (Ag)) (A) and further HI assays against other antigens: clade 2.3.4.4b (H5N1 A/chicken/Ger-SH/AI08298/2021 (Ag) (B); clade 1 (H5N1 A/chi/Viet/P41-05 (R75/05) (Ag)) (C) and Genotype 2.II (NDV Clone 30) (D)
